# Uneven Treadmill Training for Rehabilitation of Lateral Ankle Sprains and Chronic Ankle Instability: Protocol for a Pragmatic Randomized Controlled Trial

**DOI:** 10.2196/38442

**Published:** 2022-06-22

**Authors:** Elizabeth Russell Esposito, Shawn Farrokhi, Benjamin R Shuman, Pinata H Sessoms, Eliza Szymanek, Carrie W Hoppes, Laura Bechard, David King, John J Fraser

**Affiliations:** 1 Department of Defense -Veterans Affairs Extremity Trauma and Amputation Center of Excellence Joint Base San Antonio-Fort Sam Houston San Antonio, TX United States; 2 Center for Limb Loss and Mobility Veterans Affairs Puget Sound Health Care System Seattle, WA United States; 3 Department of Mechanical Engineering University of Washington Seattle, WA United States; 4 Department of Physical Medicine and Rehabilitation Uniformed Services University Bethesda, MD United States; 5 Department of Physical and Occupational Therapy Naval Medical Center San Diego San Diego, CA United States; 6 Henry M Jackson Foundation Bethesda, MD United States; 7 Warfighter Performance Department Naval Health Research Center San Diego, CA United States; 8 Madigan Army Medical Center Joint Base Lewis-McChord, WA United States; 9 Doctoral Program in Physical Therapy, Army-Baylor University U.S. Army Medical Center of Excellence Joint Base San Antonio-Fort Sam Houston San Antonio, TX United States; 10 San Antonio Military Medical Center Fort Sam Houston San Antonio, TX United States; 11 Operational Readiness and Health Directorate Naval Health Research Center San Diego, CA United States; 12 Primary Care Sports Medicine Fellowship Naval Hospital Camp Pendleton Oceanside, CA United States

**Keywords:** military personnel, ankle injuries, rehabilitation, recovery of function, secondary prevention, ankle sprain, treadmill

## Abstract

**Background:**

Lateral ankle sprains (LASs) are common injuries among military service members. Approximately 40% of individuals with an LAS progress to develop chronic ankle instability (CAI), a condition that results in substantial mechanical and neurophysiological impairment and activity limitation. Since proprioceptive and balance training improve functional outcomes and prevent secondary injury following LAS, they are recommended in clinical practice. Uneven treadmills are an innovative modality that challenge the sensorimotor system while performing an ecologically valid task simulating environments frequently encountered by service members with LAS and CAI.

**Objective:**

The aim of this study is to evaluate whether the inclusion of uneven treadmill training in standard rehabilitation can improve clinical, functional, biomechanical, and patient-reported outcomes compared with the standard of care alone in service members with LAS and CAI. The prophylactic effects of treatment on secondary injury and identification of any contributing or mediating factors that influence outcomes following treatment will also be evaluated. We hypothesize that service members receiving uneven treadmill training will demonstrate greater improvements in clinical and instrumented measures of impairment, patient-reported function, and lower risk of injury recurrence than the control group immediately post and 18 months following treatment.

**Methods:**

A multisite, parallel randomized clinical trial will be performed among service members aged 18-49 years being treated for LAS and CAI in military treatment facilities in the United States. Participants randomly assigned and allocated to receive the experimental intervention will be provided up to 12 sessions of training on an uneven terrain treadmill over a 6-week treatment course to supplement standard rehabilitation care. Treatment intensity of the rehabilitation exercises and treadmill training will be progressed on the basis of patient-perceived intensity and treatment responses. Outcome measures will include patient-reported outcomes, functional assessments, performance measures, and biomechanical measures. Investigators collecting outcome measures will be blinded to treatment allocation. Reinjury rates and patient-reported outcomes of function will be tracked over 18 months following treatment.

**Results:**

The project was funded in September 2020. Patient recruitment began in November 2021, with 3 participants enrolled as of February 2022. Dissemination of the main study findings is anticipated in 2024.

**Conclusions:**

This study will assess the impact of an innovative uneven-terrain treadmill on treatment outcomes in the rehabilitation of service members with LAS and CAI. The results of this study will be used to inform rehabilitation practices and to potentially improve functional outcomes and secondary prevention in this patient population.

**Trial Registration:**

ClinicalTrials.gov NCT04999904; https://clinicaltrials.gov/ct2/show/NCT04999904?term=NCT04999904

**International Registered Report Identifier (IRRID):**

DERR1-10.2196/38442

## Introduction

Lateral ankle sprains (LAS) are one of the most common injuries in the United States [1]. The burden of these injuries is even higher in the military, where the incidence is substantially greater in enlisted service members (21.3 to 33.4 per 1000 person-years) than in their civilian counterparts (19.0 to 26.6 per 1000 person-years) [1,2]. LAS substantially degrade the ability of the military to meet operational objectives, with an average of 14 days of lost duty time per injury [3] and more than 92,000 medical visits for the care of these injuries incurred per year [4]. While the burden of LAS determined by historical medical encounters is high, the true burden is likely much higher. Perceptions of LAS as a benign and self-limiting condition preclude care-seeking by service members [5]. Moreover, injury recurrence is common during the first 12 months following injury [6,7] and beyond [8]. Approximately 40% of individuals with LAS are projected to progress to develop chronic ankle instability (CAI) [8,9]—a complex clinical entity characterized by mechanical and sensorimotor impairments that result in long-term disability and degraded health-related quality of life [10].

Peripheral and central sensorimotor impairments are common in LAS and CAI. These neurophysiological deficits are the result of deafferentation from connective tissue and muscle injury, peripheral nerve injury, spinal- and cortical-level inhibition, changes in the cortical motor map, and central sensory reorganization [11]. For example, individuals with CAI demonstrate increased reliance on visual afference during dynamic tasks [12], which is a compensatory strategy to counter diminished plantar sensation [12-14]. Altered walking mechanics are also common in individuals with CAI, specifically a wider base of support, increased shank external rotation, a more plantarflexed and supinated foot, and a more laterally displaced path of center of pressure (COP) progression compared with uninjured individuals [15]. These adaptations are due, in part, to deleterious neuromotor changes in function, which affect the response time to lateral perturbations and reduce the ability to counter ankle inversion to prevent reinjury [11,15].

Based on the burden and morbidity associated with LAS and CAI, proper management of these injuries is especially salient in service members. This population has high physical demands and esoteric occupational and environmental exposures that increase injury risk [2]. Clinical practice guidelines for the treatment of LAS recommend inclusion of early progressive weight bearing, manual therapy, and functional exercises that include proprioceptive balance training [16]. These updated guidelines further recommend these exercises for secondary prevention of subsequent injury and outline the necessity for graded exposure to occupational-related tasks [16]. Proprioceptive training has been shown to improve some of the postural control deficits and COP excursions in patients with LAS and CAI [11] and to decrease the risk of reinjury [7,16]. Provision of therapeutic exercises, including proprioceptive training, early in the rehabilitation course has been shown to be prophylactic during the first 12 months following LAS [7].

Despite the demonstrated benefits of proprioceptive training and early rehabilitation following LAS, there is no evidence to suggest that the burden of CAI is decreasing. Currently, most proprioceptive training encompasses the use of static balance exercises performed on solid and compliant surfaces [17]. While static exercises are indeed important as part of a comprehensive rehabilitation course, they have limited ecological validity and may not provide the specificity needed to prepare service members for environmental hazards frequently encountered in the performance of occupational duties. Treatment modalities that provide graded exposure to perturbations during functional activity may better help to facilitate sensorimotor plasticity and integration in preparation for return to duty [11]. The purpose of this pragmatic randomized controlled trial is to determine if incorporation of graded exposure on an uneven-terrain treadmill during standard rehabilitation care will lead to greater improvement in function and secondary preventive effects than standard rehabilitation care alone in military service members with LAS and CAI up to 18 months following treatment. The secondary aim of this is to identify the factors that predict and mediate treatment outcomes in this population.

## Methods

### Study Design

This study will use a multisite, parallel randomized clinical trial, with Group (control and experimental) and Time as the independent variables. Methodological development was informed using the PRagmatic Explanatory Continuum Indicator Summary (PRECIS-2) tool [18]. The CONSORT (Consolidated Standards of Reporting Trials) statement for randomized trials of nonpharmacologic treatments [19] and the Template for Intervention Description and Replication (TIDieR) checklists [20] were used to guide reporting in this protocol.

### Recruitment

A total of 312 participants (LAS: n=156; CAI: n=156) will be recruited across 3 sites in the Military Health System (MHS), including the Naval Medical Center San Diego, California, the Naval Hospital Camp Pendleton, and the San Antonio Military Medical Center from November 2021 to approximately March 2023. Individuals who incurred a recent LAS and were assessed in the MHS will be identified using the electronic medical records and be contacted to discuss their interest in participation. Patients referred by primary care and specialty care clinics will be provided information on the study and an opportunity to participate. All treatments will be delivered in an outpatient physical therapy clinical setting.

### Inclusion and Exclusion Criteria

Individuals aged 18-49 years will be recruited to participate. Inclusion in the LAS stratum will require the occurrence of a substantial first-time sprain up to 2-6 weeks prior to obtaining consent, which limits functional activity for at least 1 day. Participants must be able to walk community distances without an assistive device and with a score of ≤4 out of 10 reported on the numeric pain rating scale (NPRS). Inclusion in the CAI stratum requires a history of at least one significant sprain greater than 12 months prior to consent, continued perceived or episodic “giving way” of the ankle, and reported decreased function on the Foot and Ankle Ability Measure (FAAM) activities of daily living (ADL) subscale (score≤90), FAAM Sports subscale (score≤80), and Cumberland Ankle Instability Tool (CAIT; score<24) [21]. Individuals will be excluded if they have previously undergone ankle surgery, had joint realignment owing to fracture, had recent (within the last 3 months) injury to other lower-limb joints, have a diagnosed connective tissue disorder (eg, Marfan syndrome or Ehlers-Danlos syndrome), have a neuromuscular disease or balance impairment (eg, visual or vestibular disorder) that precludes standing or walking, are pregnant, have nonremovable casting, or are unable to walk at enrollment.

### Ethical Considerations

The study protocol was approved by the institutional review boards at the Naval Medical Center San Diego, the Naval Health Research Center, and the US Army Regional Health Command-Central in compliance with all applicable Federal regulations governing the protection of human subjects. Research data were derived from an institutional review board–approved Naval Medical Center San Diego protocol (NMCSD.2020.0028). All participants will be required to provide informed consent prior to study enrollment. Recruitment of military service members will occur in the absence of supervisory staff to avoid any perceived coercion. Participants will also be free to withdraw at any time during the study. Because this study is being conducted within the MHS, all participants who decline to participate in this research will still be provided standard rehabilitation care.

### Intervention

#### Overview

Once consented and enrolled, participants will be randomly assigned to either the experimental or control arm at each site using a concealed, random blocked sequence. Stratified randomization will be used to ensure an equal number of men and women are assigned to both the control and experimental groups in the LAS and CAI strata. Group allocation information will be concealed using a password-protected electronic form that will be opened by an uninvolved research staff member and communicated to the treating clinician. Both experimental and control groups will be provided standard rehabilitation care—treatment that is guided by the recent revision of the clinical practice guideline promulgated by the Academy of Orthopaedic Physical Therapy [16] on the basis of clinician experience and patient values.

**Figure 1:**
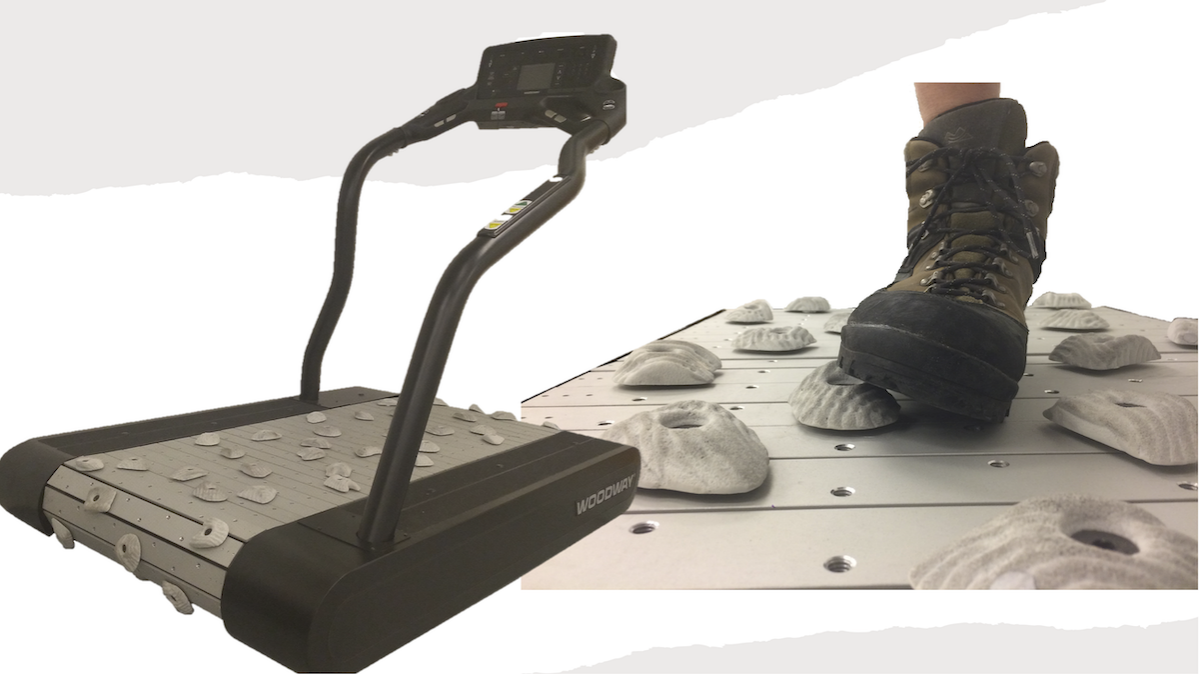
The custom uneven-terrain treadmill. Participants allocated to the experimental treatment will be provided a progressive gait retraining program in addition to standard rehabilitation care.

Participants allocated to the experimental group will also be provided a complementary progressive treatment under the supervision of a licensed physical therapist, consisting of walking on a custom uneven-terrain treadmill (Woodway, Inc) (Figure 1) that simulates negotiation of rocky terrain (Multimedia Appendix 1) [22] for approximately 30 minutes, twice weekly, for a maximum of 12 treatments until discharge from rehabilitation. Treatment will be advanced through 3 phases of activities based on observed performance and the participant’s perceived difficulty and symptom response. Each phase will contain activities that are broadly grouped by the intensity of the proprioceptive stimulus and will be completed on the uneven treadmill. All treating physical therapists will adhere to a single training protocol to ensure standardized delivery of the uneven treadmill training across all the participating sites.

#### Experimental Treatment Progression

##### Phase 0: Familiarization

Participants will begin a familiarization trial during which they will walk on the treadmill at a low velocity (≤1.34 m/s) set at a level grade for at least 5 minutes while wearing an ankle brace. Continued use of the handrails for balance and offloading the injured limb will be allowed during this phase. Criteria for progression will depend on the symptom response, patient confidence, and the observed ability to walk unassisted.

##### Phase 1: Unloaded Treadmill Walking

Walking velocity will be progressively increased, and the ankle brace will be weaned on the basis of the pain response and perceived instability. As the participant acclimates to the activity, task difficulty will be incrementally progressed by manipulating the walking velocity, grade, and duration in accordance with patients’ perceived difficulty and performance observed by the treating clinician.

##### Phase 2: Unloaded Treadmill Walking With Head Movement and Visual Manipulation

Building on phase 1, the purpose of this phase is to encompass the integration of head movement, visual occlusion, and distraction. The following conditions will be integrated to increase task difficulty, as appropriate:

1. Low light condition: dark sunglasses (Sunglass Couture, Inc) will be used to reduce the amount of ambient light.
2. Foot obscurement: foot placement will be obscured with dribble glasses (Liberty Imports) that obstruct participants’ view of the ground while carrying an inert training rifle (Ring’s Manufacturing Inc).
3. Head turning: during the head turning activity, participants will be instructed to walk while turning their head right, left, upward, and downward.
4. Distracted walking: during the distracted walking activity, participants will walk while playing a handheld electronic game (Classic 5 In 1 Poker, John N. Hansen Co, Inc).

##### Phase 3: Return to Duty and Recreational Running Tasks

The criteria for progression to this phase are met when the participant begins the transition from walking to running activities and has a FAAM ADL subscale score of >90 and FAAM Sports subscale score of >80.

1. Load carriage: the integration of a 12-kg load carried in a backpack (SOG Specialty Knives & Tools) will be included in phase-1 and -2 activities.
2. Uneven trail running: integration of jogging (<2.78 m/s) and running (≥2.78 m/s) may be optionally performed in preparation for return to duty or recreational running.

### Outcome Measures

**Figure 2:**
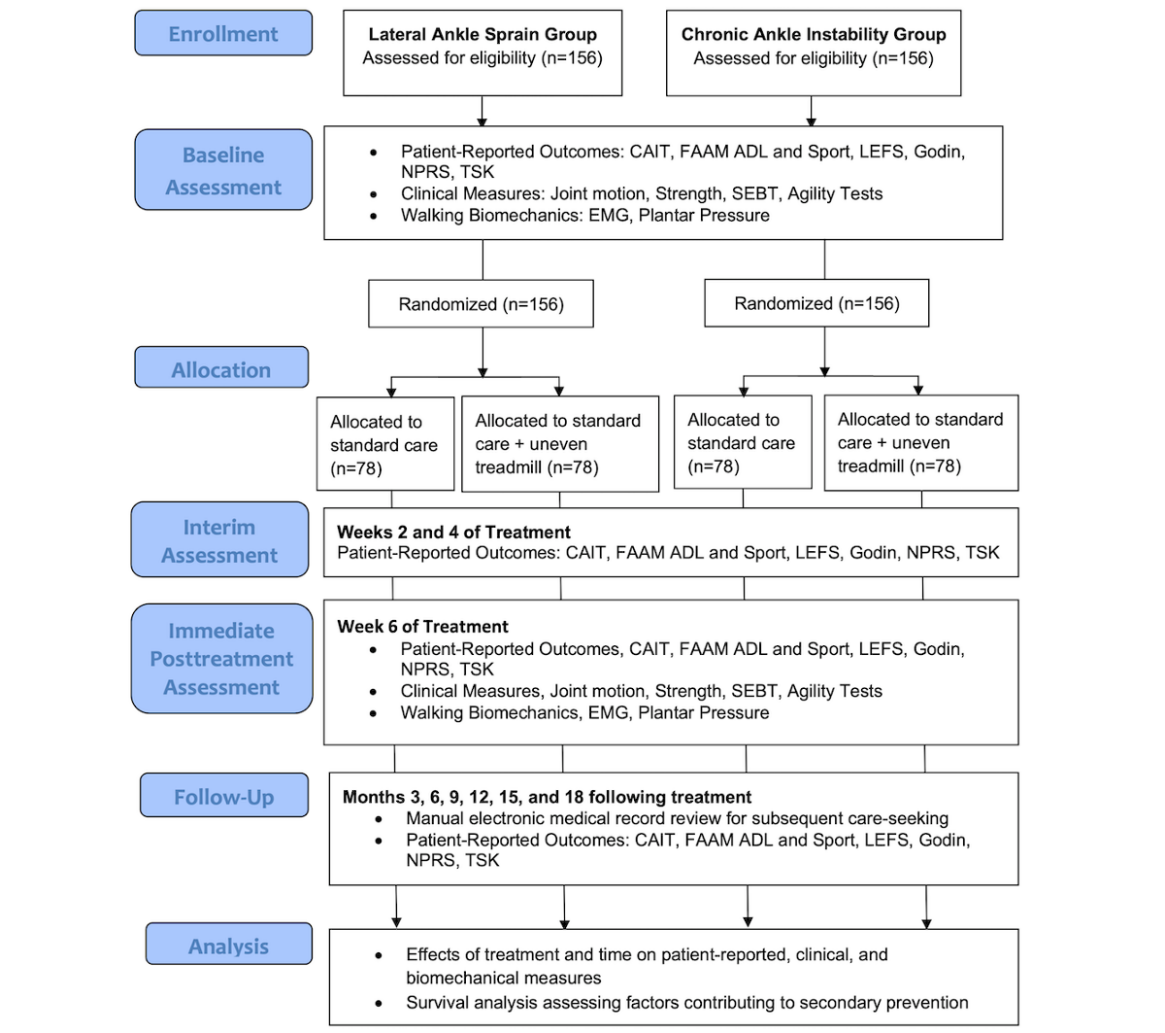
The CONSORT (Consolidated Standards of Reporting Trials) flow diagram. ADL: activities of daily living subscale, CAIT: Cumberland Ankle Instability Tool, EMG: electromyography, FAAM: Foot and Ankle Ability Measure, Godin: Godin-Shepard Leisure-Time Physical Activity Questionnaire, LEFS: Lower Extremity Functional Scale, NPRS: numeric pain rating scale, SEBT: Star Excursion Balance Test, TSK: Tampa Scale of Kinesiophobia.

A series of clinical, instrumented, and patient-reported outcome measures will be collected to characterize ankle-foot impairment, activity limitation, and participation restriction at baseline, during treatment, and longitudinally following treatment. Figure 2 shows the CONSORT flow diagram that details the study time points. The primary outcome of interest is improvement in patient-reported function on the FAAM. To help elucidate clinical improvement in function, we will assess if the intervention has shifted COP progression more medially during walking and if recurrence of subsequent ankle injuries has been reduced. Reinjury count data will be collected and assessed in both groups.

### Patient-Reported Outcomes

The CAIT [23] is a 9-question instrument examining ankle pain and feelings of instability. The FAAM ADL and Sports subscales [24,25] and the Lower Extremity Functional Scale [26] assess difficulties in performing a variety of tasks. The NPRS [27] and Tampa Scale of Kinesiophobia [28] will be used to assess the participants’ pain and fear of movement due to the injury. Physical activity will be classified using the Godin-Shepard Leisure-Time Physical Activity Questionnaire [29] modified to record activity over the previous week rather than a typical week. These patient-reported outcomes will be recorded at all data collections (baseline, 2 interim sessions, discharge, and 6 postintervention follow-up assessments). Unstructured feedback requesting perceptions on participation in the uneven treadmill rehabilitation protocol will also be collected.

### Clinical Measures

Clinical measures of ankle dorsiflexion (unloaded and loaded), plantarflexion, inversion, and eversion; forefoot on rearfoot inversion and eversion; and strength measures of dorsiflexion, plantarflexion, inversion, eversion, and hallux and lesser toe flexion will be performed, as described by Fraser et al [30]. The Star Excursion Balance Test evaluates dynamic balance and control by asking the patient to reach as far as possible along a measuring tape affixed to the floor in anterior, posteromedial, and posterolateral directions while maintaining balance on the supporting foot [31,32]. Failure to maintain balance, hands on the hips, or weight shift onto the reach limb will constitute a mistrial and will be repeated. Agility, speed, and power will be assessed using the side hop test [33], Edgren Side Step Test [34], and the T-test [34]. The side hop test records how many times a patient can hop back and forth on one limb over a distance of 30 cm in 30 seconds [33]. The Edgren Side Step Test has participants side-step back and forth over a 4-m course for 10 seconds [34]. The T-test measures the time to run 40 m multidirectionally over a T-shaped course [34]. Participants will be provided familiarization of the tests and practice trials prior to collection. Verbal encouragement will be provided during assessments.

### Biomechanical Measures of Walking

Electromyography (EMG) and plantar pressures will be collected while walking on a standard treadmill at a self-selected velocity. Plantar pressure data will be recorded at 50 Hz using a Pedar-X shoe insole (Novel Electronics, Inc). EMG data (Trigno Avanti, Delsys Inc, and Noraxon DTS) will be recorded at 1500 Hz or greater bilaterally using Ag/AgCl electrodes affixed to 6 muscles (tibialis anterior, fibularis longus, lateral gastrocnemius, rectus femoris, biceps femoris, and the gluteus medius). Electrode placement will be performed as described by Weiss et al [35]. Visual inspection of the waveform during resisted contraction of each muscle will ensure cross talk is minimized. EMG data collected during walking will be processed by (1) using a fourth-order Butterworth filter for band-pass filtering between 20 Hz and 400 Hz, (2) rectifying the data, and (3) smoothing using a fourth-order Butterworth low-pass filter at 10 Hz and normalized to quiet standing. To compare walking EMG data between sessions, the amplitude will be normalized to the mean root mean square of processed EMG data collected during quiet standing for each muscle [36,37]. Gait cycles will be identified using a force-sensitive resistor located under the heel to detect heel strike. Data from at least 15 consecutive strides will be collected during steady-state walking.

### Longitudinal Tracking of Reinjury

Injury count data derived following completion of the intervention will be employed to assess secondary preventive effects of the control and experimental treatments in the LAS and CAI strata. These follow-up data will be collected for 18 months. Both in-network and out-of-network medical care provided to service members will be captured by the MHS. Each recruiting site will conduct a chart review of the electronic medical record at the 6 posttreatment time points to assess for any additional medical care provided for ankle injuries in order to include physical therapy or surgical care. Participants will also be contacted directly to establish any secondary injury outcomes that were self-managed.

### Blinding

Any research staff collecting data at any time point will be blinded to group assignment. Owing to the nature of the intervention, blinding of the participants or the clinicians providing the uneven-terrain treadmill rehabilitation intervention will not be feasible. Data analysis will be performed by researchers and statisticians blinded to group assignments.

### Statistical Analysis

To assess whether the addition of the rocky treadmill intervention to standard-of-care physical therapy improves rehabilitation, 4 repeated measurements of several outcomes (eg, CAIT and FAAM) will be measured over the active intervention period of the study. The data will be analyzed using an analysis of covariance–type linear mixed-effects model with Group, Time, and Group×Time variables as fixed effects and subject-specific variables {intercepts, slopes} as random effects. The null hypotheses for both LAS and CAI populations are that the slopes of the control groups equal those of the treatment groups.

**Figure 3:**
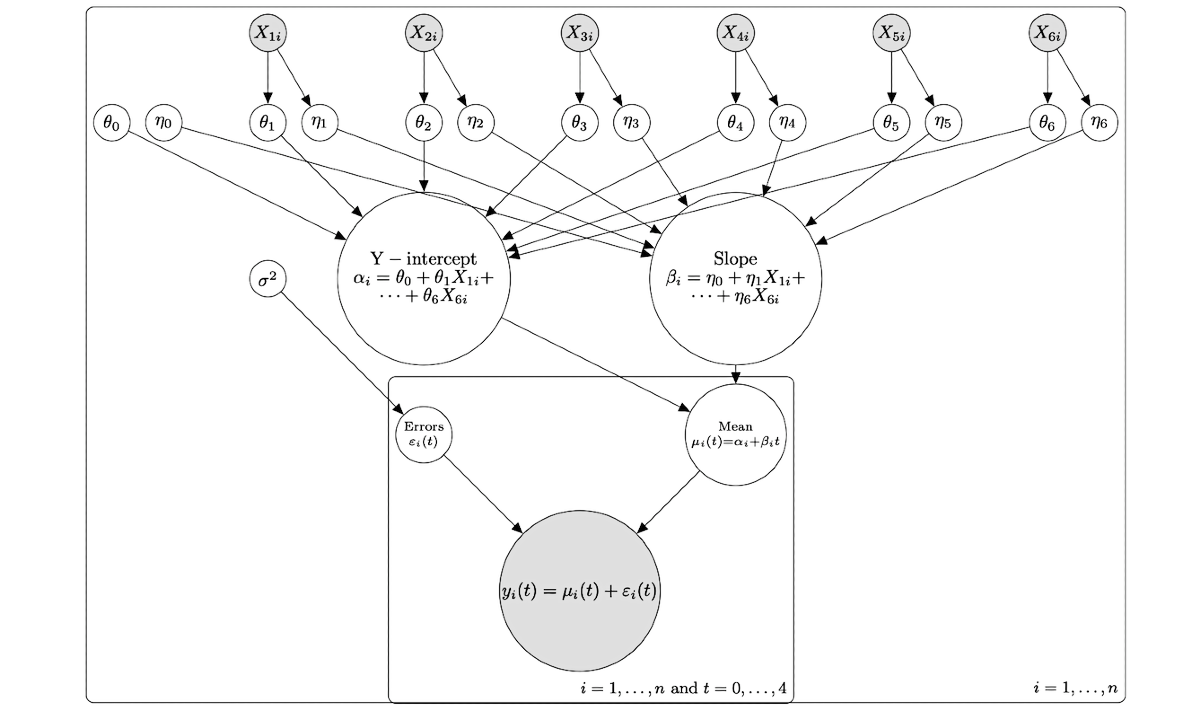
The direct acyclic graph of the Bayesian model acts like a causal diagram. In the model, the explanatory variables {X1, . . . , X6}: age, sex, BMI, pain intensity, weight-bearing status, and initial injury severity. These variables causally affect the subject-specific slopes and the y-intercepts. However, the slopes and y-intercepts of the responses are not directly observed, so they are considered latent variables. In the diagram, the shaded circles are directly observed variables, and the unshaded circles are latent variables. The Bayesian model has 2 levels of hierarchy: the first one establishes the best fit line that goes through the observed subject responses; the second one establishes how the explanatory variables {X1, . . . , X6} affect the slopes and y-intercepts. The regression parameters {η_0_, . . . , η_6_} are those that affect the slope of the subject responses and, as such, are particularly important because they affect the healing rate. Variables that significantly affect the healing rate will be retained in the model if the credible intervals for the regression parameters {η_0_, . . ., η_6_} do not include 0.

To identify predictors and mediators of successful or unsuccessful rehabilitation outcomes, a 2-level (hierarchical) Bayesian model will be used to connect participant-specific responses to 6 explanatory variables: {X1, . . . , X6} = {age, sex, body mass index, pain intensity, weight-bearing status, and initial injury severity} to the subject-specific variables {intercepts, slopes}. A directed acyclic graph detailing the results of the Bayesian linear model will be developed (Figure 3). The regression parameters from the hierarchical Bayesian model will be estimated using Bayesian inference with Gibbs Sampling software, which creates a Markov chain Monte Carlo simulation of all regression parameters.

To analyze the effect of the treatment on long-term reinjury rates between the 2 arms of the study, we will fit both logistic regression–type generalized linear mixed-effects (GLME) models to the data as well as Cox regression time-to-event models. The data that will be used in the analysis will be those obtained at baseline and during the postintervention period. In the GLME analysis, the response data will be (1) the self-reported survey instrument measure from the Patient-Reported Outcomes Measurement Information System and (2) the binary reinjury report (1/0). The GLME model will have Group, Time, and Group×Time as fixed effects and will use subject-specific variables {intercepts, slopes} as random effects. The null hypothesis of the GLME model is that the regression parameters associated with the Group and Group×Time effects are zero. In the Cox regression analysis, the response will be the time of suspension or the event along with a binary indicator of reinjury status, and the covariate will be Group. The null hypothesis of the Cox regression analysis is that the regression parameter associated with Group is equal to zero. In all analyses, a Bonferroni-type correction to the hypothesis test’s *P* values or α levels will be used to account for multiple testing.

### Power and Sample Size

The estimated sample to identify a minimum difference of 1 point in the FAAM between treatment groups across 4 time points in the intervention period was 17 participants per group for an estimated power of 90% and an α value of .05. To measure the effect of the long-term binary reinjury rates with 6 time points in a GLME model, a sample of 62 participants per group provided 80% power, assuming a 10% difference in the proportion between the treatment groups and similar trends between groups. Assuming a 20% rate of dropout throughout long-term follow-up, the required sample size is 78 participants per group. Between the 2 injury types (LAS and CAI), a total of 312 participants will be recruited for this study across the multiple sites.

## Results

Data collection began in fall 2021. Dissemination of the main study findings is targeted for 2024.

## Discussion

This pragmatic randomized controlled trial will assess the clinical effects of an innovative uneven-terrain treadmill in the rehabilitation of patients with LAS and CAI. We hypothesize that service members receiving uneven treadmill training will demonstrate greater improvements in clinical and instrumented measures of impairment, patient-reported function, and lower risk of injury recurrence than the control group immediately post and 18 months following treatment.

Integration of an ecologically valid activity that replicates negotiation of uneven, rocky ground commonly encountered by the military population may facilitate adaptive improvements in sensorimotor function and a return to occupation and recreational activities and may help prevent secondary injuries. Uneven-terrain treadmills present the opportunity to progressively increase the rehabilitation stimuli by incorporating tasks essential to military performance, including walking with a weapon (which obstructs the downward view of foot placement) and load carriage (which has been shown to reduce dynamic stability) [38]. Uneven-terrain treadmills also offer a relatively low-cost platform for progressive proprioceptive training, simulating real-world terrain that challenges ankle stability. The pragmatic nature of this study ensures that standard-of-care treatment is based on the specific needs of the individual patient and is consistent with the principles of evidence-based practice. This also allows for patients assigned to the intervention group to progress to more challenging rehabilitation activities on the uneven treadmill based on incremental improvements in observed findings and patient response to treatment. While a limitation to this approach is the potential for differential bias in treatment dosage, a variable that will be considered and controlled for during analysis, we view this approach to be especially salient in the evaluation of clinical effectiveness of the experimental intervention that will improve the external validity of our findings.

This clinical trial will help determine if incorporation of graded exposure on an uneven treadmill during standard rehabilitation care will lead to greater improvement in function and secondary preventive effects compared with standard rehabilitation care alone in service members with LAS or CAI up to 18 months following treatment. The incorporated patient-reported pain, kinesiophobia, physical activity, and ankle function, along with clinical and biomechanical measures, will help elucidate the clinical effectiveness of incorporation of uneven treadmill training on near- and long-term function, reinjury rates, and factors that may drive treatment outcomes. Findings from this work have the potential to directly impact the care of service members, veterans, and the broader civilian community with LAS and CAI. The findings of this study will be used to generate knowledge products that will include reports, clinician and patient education materials, and presentations that will be provided to MHS clinicians, leaders, and policy makers. In addition, the findings of this study will be promulgated as peer-reviewed conference abstracts and journal manuscript submissions.

## Appendix

Multimedia Appendix 1 Supplemental video [22]. Reused under CC0 license.

Multimedia Appendix 2 Peer-review report by the U.S. Army Medical Research and Development Command - Congressionally Directed Medical Research Programs - Fiscal Year 2019 Peer Reviewed Orthopaedic Research Program - Clinical Trial Award - Funding Level 1 - Office of the Assistant Secretary of Defense for Health Affairs.

## Abbreviations

ADL: activities of daily living
CAI: chronic ankle instability
CAIT: Cumberland Ankle Instability Tool
CONSORT: Consolidated Standards of Reporting Trials
COP: center of pressure
EMG: electromyography
FAAM: Foot and Ankle Ability Measure
GLME: generalized linear mixed-effects
LAS: lateral ankle sprain
MHS: Military Health System
NPRS: numeric pain rating scale
PRECIS-2: PRagmatic Explanatory Continuum Indicator Summary
SEBT: Star Excursion Balance Test
TIDieR: Template for Intervention Description and Replication

## Disclaimer

The authors are military service members or employees of the US Government. This work was prepared as part of their official duties. Title 17, USC §105 provides that copyright protection under this title is not available for any work of the US government. Title 17, USC §101 defines a US government work as one prepared by a military service member or employee of the US government as part of that person’s official duties.

## Data Availability

The data sets generated during and analyzed in this study are available from the corresponding author (JJF) on reasonable request.
