# Supplementary material for: Uneven Treadmill Training for Rehabilitation of Lateral Ankle Sprains and Chronic Ankle Instability: Protocol for a Pragmatic Randomized Controlled Trial
Source: JMIR Res Protoc. 2022 Jun 22;11(6):e38442. doi: 10.2196/38442 (PMC9260521; doi:10.2196/38442)
Supplement: Multimedia Appendix 2 [file resprot_v11i6e38442_app2.pdf]

Department of Defense
U.S. Army Medical Research and Development Command
Congressionally Directed Medical Research Programs
Fiscal Year 2019 Peer Reviewed Orthopaedic Research Program
Clinical Trial Award - Funding Level 1
Peer Review Summary Statement

CDMRP Log Number: OR190067
Grants.gov ID Number: GRANT12937246
Review Panel: Clinical Trial
Discussion Period: 11/06/2019-11/07/2019

Project Duration: 48 months
Budget Requested: $2,429,368
Direct Costs: $2,154,467
Indirect Costs: $274,901

Title: A Proprioceptive Training Program Using an Uneven Terrain Treadmill for Patients With Ankle Instability
Principal Investigator: Elizabeth Russell Esposito
Performing Organization: Madigan Army Medical Center
Contracting Organization: Henry M. Jackson Foundation

OVERVIEW

The Principal Investigator (PI) of this application proposes to restore and improve short-term function and performance and reduce the long-term risk of reinjury for retention on duty by incorporating real-world and militarily relevant aspects of moving on uneven terrain into the safe, controlled physical therapy setting to improve outcomes following destabilizing lower limb injury. The specific aims are (1) to compare the effectiveness of a targeted proprioceptive and physical rehabilitation intervention against standard-of-care physical therapy for destabilizing ankle injuries, (2) to identify the predictors and mediators of clinical benefits and successful outcomes, and (3) to compare long-term outcomes and reinjury rates. A parallel randomized treatment clinical trial is proposed. The intervention is standard-of-care physical therapy augmented with a progressive proprioceptive rehabilitation and training program that incorporates walking tasks on a rocky, uneven terrain treadmill.

	Average Score	Standard Deviation
**Overall Evaluation**<br>*Rating Scale: 1.0 (highest merit) to 5.0 (lowest merit)*	1.9<br>**(Excellent)**	0.4
**Criteria**<br>*Rating Scale: 10 (highest merit) to 1 (lowest merit)*	**Average Score**	
**Research Strategy**	6.5	
**Intervention**	7.0	
**Recruitment, Accrual, and Feasibility**	7.7	
**Military Benefit and Clinical Impact**	9.3	
**Statistical and Data Analysis Plan**	6.4	
**Ethical Considerations**	7.6	
**Regulatory Strategy and Transition Plan**	7.4	
**Personnel and Communication**	6.8	

Procurement Sensitive Document
Do not copy or distribute without CDMRP written permission.
Prepared by SRA International, Inc., A GDIT Company, a support contractor to CDMRP

SCORED CRITERIA

Research Strategy
Average Score: 6.5

Scientist Reviewer A

Strengths: While ankle sprains are not the most traumatic injury that occurs in service personnel, the applicants provide evidence that this injury is prevalent and causes loss of force readiness and therefore is an important problem to be addressed. The investigators argue that in this patient population low visibility due to carrying weapons compounds the problem.

Evidence is presented that altered motor control and sensory deficits occur following joint injury (ie, lateral ankle sprain). Mechanical changes at the joint can further predispose these patients to subsequent injury. Sensory loss results in increased reliance on visual input, but visual input is limited due to carrying weapons. Psychological issues (kinesiophobia) contribute. This section is well written and demonstrates how this type of injury differs in the military population as compared to the general population.

The outcome measures (collected every 2 weeks/3 months) are for the most part related to function and seem appropriate for a clinical trial of this nature. The written outcome tools are standard tools for this condition. The Foot & Ankle Ability Measure seems very appropriate as the primary outcome due to its survey of a large number of functional activities, and it should be sufficient to capture change over time and between groups.

Statistical analysis is well described and seems adequate.

Inclusion and exclusion criteria are based off the International Ankle Consortium's 2014 position paper for patients with chronic ankle instability, which seems appropriate. For first-time injured, the criteria are explicitly detailed and seem appropriate.

Weaknesses: The investigators propose that their intervention will address proprioceptive deficits; however, no measures of proprioception are taken. The term is loosely used in the clinic setting, but evidence that proprioception changes following interventions is variable. It could be that the intervention will improve various strength measures, and because of that function and confidence are improved. Perhaps a better goal of this study is improvement of motor control at the ankle or a measure of balance. However, this is a minor weakness and does not affect the quality of the application.

Further information on the biomechanical measures is warranted. It is unclear where these measures are taken with shoes on or off. If shoes off, that may not replicate real-life situations. With shoes on, it is unclear whether they will make effort to ensure that all subjects wear the same type of shoes. It is not clear who will collect data at each site. While it can be assumed the physical therapists will be trained and are qualified to collect clinical measures, it is not clear whether certain personnel will be trained to collect biomechanical measures.

The study will track reinjury rate. It might also be appropriate to specifically track injury rate during use of the ruggedized treadmill—it is possible that reinjury will occur during the intervention.

Scientist Reviewer B

Strengths: There are many strengths of this proposal, including the well-justified need of the study, the expertise of the investigative team, access to the patient population of interest, use of verified data

collection procedures and outcomes, and acknowledgment of anticipated potential problems and mention of alternative approaches. In addition, the data management and sharing plan is adequate.

Weaknesses: There were several major weaknesses noted in the proposal.

The PI indicates to utilize both patients with lateral ankle sprain (LAS) and chronic ankle instability (CAI) in the study, but it is not clear if the PI hypothesizes to find more similarities or differences between the groups.

The proposed study design is a parallel, randomized controlled trial; however, it is not clear on who will randomize the patients to either the experimental group or control group and, further, if there will be equal number of patients with LAS and CAI in those groups. The recruitment distribution among the 4 sites is missing, raising doubts on the successful completion of the study.

No minimal physical activity level is required for participant enrollment, and that may influence the study results. Specifically, the results on the sensorimotor outcome measures may be affected.

The subject numbers mentioned in the abstract and proposal show a discrepancy (58 vs 78).

The inclusion and exclusion criteria are not clear and could be further strengthened. For example, a CAIT score of less than 24 is revised in the current literature, it is not clear what “chronicity of 2-6 weeks prior to consent” means, and exclusion criteria should address vision and sensorimotor issues as well as weight-bearing ability before recruiting patients in the study.

Certain details mentioned in the preliminary work are more of information than the work that sets the strong base for this proposed study.

The sophistication of the study design for Aims 2 and 3 is lacking. It is not clear on why certain outcome measures are used under the physical performance tests and biomechanical assessments.

Further, rationale on the variables used in Aim 2 is missing, and Aims 2 and 3 could be strengthened by adding the other variables that are measured in the study. More information is needed regarding the clinical trial (consider intention to treat, handling missing data, compliance, tolerance of the intervention, how ankle sprains during the intervention will be handled).

Biostatistician Reviewer

Strengths: The research design is generally appropriate for the study objectives. The data collector is blinded to the treatment allocation, but the participant is not. Due to the intervention at hand, it is likely not possible to blind the participant to the treatment. The outcomes have good psychometric properties.

Weaknesses: Specific Aim 2 is not related to evaluating the intervention, such as determining if responders/nonresponders are more likely to be in the treatment or control group. It is not clear why 4 measurements of the primary and secondary outcomes are recorded if the test-retest reliability of these measures is so high. An 18-month follow-up risks dropout at much larger than 20% for Specific Aim 3.

Discussion Notes

Reviewers expressed different levels of enthusiasm for the research strategy. Some thought that the ruggedized uneven treadmill is a novel type of intervention that mimics real-life conditions much better than other, more static designs. They also thought that whereas there has been lots of research on physical

Procurement Sensitive Document
Do not copy or distribute without CDMRP written permission.

therapy to rehabilitate the knee, not much has been done for the ankle, and thus the rationale for the study is compelling. Some reviewers were concerned that measures of proprioception and some biomechanical measures are not well developed. However, others were less concerned as this is a clinical trial with functional clinical outcomes, not a preclinical study looking at mechanisms.

Intervention
Average Score: 7.0

Scientist Reviewer A

Strengths: All study participants will receive standard-of-care physical therapy over the 6-week time frame, and the experimental group will also receive training on the ruggedized treadmill. Physical therapists are allowed to include a variety of interventions as a part of the standard-of-care treatment; however, the investigators have limited inclusion of balance activities on unstable surfaces (such as a BOSU). It is understandable why they made this limitation; however, the assumption to be taken from this application is that walking on the ruggedized treadmill will provide benefit above and beyond typical training on basic unstable surfaces. Considering Gentile's Taxonomy of Tasks, the ruggedized treadmill is at the highest complexity considering body stability, environmental condition, and body transport. Progression on the ruggedized treadmill intervention is well described, and a pragmatic approach to progression seems appropriate.

Weaknesses: It might be questioned whether the basic intervention is standard of care without training on unstable surfaces. One potential concern is that the criterion that triggers progression between stages could be more objective.

Scientist Reviewer B

Strengths: The rehabilitation strategy is rooted in evidence. The brief overview of the 6-week targeted proprioceptive and physical rehabilitation intervention and standard-of-care physical therapy is provided. The time frame of the exercise protocols as well as data collection on various outcome measures seems feasible. The proposed rehabilitation strategy can be administered in any outpatient clinical setting and is considered safe to administer.

Weaknesses: Though the activities under each rehabilitation phase/level are mentioned in the proposal, the rationale for exercise progression and dosage under each rehabilitation protocol is missing. The explicit criterion to progress the patient to a higher level is missing.

Recruitment, Accrual, and Feasibility
Average Score: 7.7

Scientist Reviewer A

Strengths: Recruitment is well described. The investigators have experience in these type of trials and describe a plan to mitigate dropout from the study.

Weaknesses: One small issue, likely a typo, is that the abstract identifies 58 subjects in each group while the main application identifies 78 subjects in each group.

Procurement Sensitive Document
Do not copy or distribute without CDMRP written permission.

Scientist Reviewer B

Strengths: The participating sites (VA Puget Sound Health Care System, Madigan Army Medical Center, Naval Health Research Center, and Naval Medical Center San Diego) should not have difficulty recruiting patients with LAS and CAI. The PI has obtained necessary letters of support. The lab space and equipment in collaborating institutions appear adequate to carry out the proposed study. The investigators have adequate access to patients with LAS/CAI and have extensive experience in key areas of clinical trial management. Participation in this study should not affect the daily lives of the participants.

Weaknesses: The regular meetings with the site PIs are mentioned; however, the discussion on contingency plans to address slow accrual or minimize attrition is missing.

Military Benefit and Clinical Impact
Average Score: 9.3

Scientist Reviewer A

Strengths: The study has the potential to provide important benefits in terms of force readiness. The investigators potentially also address prevention of repetitive injury and the progression of chronic ankle instability. This not only has potential benefit for the military but also for the general population.

Weaknesses: No weaknesses were noted.

Scientist Reviewer B

Strengths: The proposed study aligns with FY19 CDMRP PRORP Clinical Trial Award focus area of retention on duty strategies. Specifically, this study addresses the rehabilitation strategies designed to "train to the task" to prepare individuals to return to the activities they will encounter in their occupation or sports setting.

Considering that ankle sprains are very common in military personnel, the new rehabilitative and preventive strategies developed as a result of this proposal can be incorporated into current training programs for active military personnel to prevent recurrent ankle injuries. If successful, the device would be useful in treatment of sports injuries. There is potential for immediate and long-term effect/benefit and usability of the proposed research on the health and well-being of active service members.

Weaknesses: There is limited support in the background section of the application that the intervention will be successful and ultimately lead to having an impact for military service members as well as veterans.

Consumer Reviewer

Strengths: This proposal has significant benefits for addressing the issue of ankle injuries and lost time. Even though an individual is more prone to have another ankle injury and recurrence increases with each additional injury, this addresses not only healing but actual retraining. As cited in the proposal, the benefits of a CAREN system are the premier standard, but due to cost it will never be implemented, and this provides a low-cost alternative that can even be moved into a forward operating base (FOB) in a combat environment. Moving this style of treatment, being so close to point of injury, reduces the need to evacuate a soldier, allows the service member to recover while still being able to perform alternate tasks in a FOB, and is a noninvasive treatment.

Procurement Sensitive Document
Do not copy or distribute without CDMRP written permission.

Weaknesses: No weaknesses were noted.

Discussion Notes

Reviewers were excited about the proposed ruggedized treadmill training; they saw it as a relatively inexpensive tool that can be used in many sites, including close to point of injury and forward operation locations as well as many settings relevant to veterans. Reviewers expect real buy-in from injured soldiers to enable them to return to duty. Thus, the proposed treatment has very clear military application and is easily transferrable if research aims are met.

Statistical and Data Analysis Plan
Average Score: 6.4

Scientist Reviewer A

Strengths: Statistical analysis is well described.

Weaknesses: Analysis of the biomechanical measures is not described. How these data will impact the specific aims of this study is not clear.

Scientist Reviewer B

Strengths: A generic statistical plan for Aims 2 and 3 is presented.

Weaknesses: The statistical plan could be more individualized for the proposed study. The sample size projection and power analysis look adequate for the proposed study, though the assumption made to calculate empirical power is unclear.

Biostatistician Reviewer

Strengths: Regarding all specific aims, all planned analyses are appropriate to assess the proposed aims. Repeated measurements over time are properly incorporated in the statistical analysis plan.

Regarding Specific Aim 2, the study team should be commended for proposing a rather complex, nonstandard analysis for assessment of this aim.

Regarding Specific Aim 3, an adjustment for multiple comparisons is a conservative approach for secondary outcomes.

Primary outcome is assessed at or below the minimal clinically important difference (MCID).

An adjustment for 20% lost to follow-up is included.

The proposed sample size is moderate/large, which should aid in detecting clinically relevant effect sizes and permit the analysis in all specific aims.

Weaknesses: Regarding all specific aims, there is no mention of the appropriateness of the statistical analysis methods in the presence of missing data, particularly if it is related to response/nonresponse. The data analyses depend heavily on a linearity assumption, which may not be reasonable, especially if the outcomes plateau prior to the end of the time frame. There are no planned assessments or adjustments for differences in the study strata (acute ankle sprain, chronic ankle instability) or by site. The latter omission in this effectiveness study is particularly important as “standard of care” may differ between sites.

Procurement Sensitive Document
Do not copy or distribute without CDMRP written permission.

Regarding Specific Aim 1, there is no adjustment for taking 4 measurements of each outcome at each time point. Without careful handling of these measurements, the power of this test resulting from this model may be artificially inflated.

The sample size justifications for Aims 1 and 3 are not reproducible. It is impossible to determine if the statistics presented are valid without key pieces of information, such as parameters used for the Aim 1 sample size justification and the baseline rate for the Aim 3 sample size justification.

Ethical Considerations
Average Score: 7.6

Scientist Reviewer A

Strengths: Safety monitoring is well described. This proposed intervention has great potential as a rehabilitative tool.

Weaknesses: One potential concern is that the criterion that triggers progression between stages could be more objective.

Scientist Reviewer B

Strengths: The patients enrolled in both the rehabilitation protocol groups would benefit from participating in the study. If the progressive proprioceptive rehabilitation and training program is found to mitigate the risk of recurrent injury and improve long-term outcomes, there is potential to affect the CAI rehabilitation paradigm. Licensed physical therapists will be conducting the rehabilitation at the 4 testing facilities. The proposed rocky terrain treadmill is available through a commercial vendor. Adequate steps to seek informed consent/minimize the level of risk/protect privacy and confidentiality are described in the proposal or the supporting appendixes.

Weaknesses: A clear progression criterion to higher-level activities in the progressive proprioceptive rehabilitation and training program and harness use during the dynamic training will minimize the risk of ankle reinjuries.

Bioethicist Reviewer

Strengths: The exclusion criteria are justified scientifically and consistent with the International Ankle Consortium recommendations. Risks are well described, and the risk mitigation plan addresses those known potential risks. The recruitment of subjects is well described and, in general, well thought out. The remuneration schedule is clearly spelled out in the consent form. Due to the presentation of the schedule, it is assumed that payment will be for completion of tasks, such that a prorating schedule is not required.

Weaknesses: Exclusion of participants more than 49 years of age who can experience ankle instability without scientific justification excludes a portion of the population who stand to benefit from the knowledge gained and thus may be a violation of the Justice principle. While the exclusion of pregnant women is justified, there is no indication that pregnancy testing will be performed, it is not mentioned in the consent form, and there is no indication of what will happen to the subject's continuing participation should the subject become pregnant during the 18 months of study participation. The protocol describes that one benefit is that subjects in the intervention group will receive specialized training as part of the study. This may or may not be a benefit, and that will not be known until the study is complete, as this is the question that the study proposes to answer. In addition, remuneration for time is not to be considered a benefit. The protocol states that the study team will record all potential adverse and safety events on the

Procurement Sensitive Document
Do not copy or distribute without CDMRP written permission.

"consent form attendance log." It is not clear what this log is, but it does not seem an appropriate place to record adverse events. The protocol is inconsistent concerning an independent medical research monitor. In one place it states that this will occur only if the IRB determines that the study is greater than minimal risk; in the Clinical Monitoring Plan section, it states that an independent medical research monitor will be included at each study site. The qualifications of a medical monitor are not described, and a person is not named. Monitoring is focused on protocol adherence rather than safety. The data management section appears to indicate that the subject ID (well described) would break the blind—that it can be determined from the ID which group the subject is randomized to. Recruitment methods include that there will be some number of potential subjects who will receive blind approaches, not introduced by a clinician they know, and without the potential subject having indicated permission to contact. This should not occur. The protocol (data management section) mentions video and photography, but any discussion of protections to privacy/confidentiality of films is absent in the consent form. The protocol (data management section) mentions that subjects can obtain a copy of their own study data, but this is absent in the consent form. Key information at the beginning of the consent form is missing; this requirement became effective in January 2019. If standard of care includes a wobble board and that standard of care is being withheld from research subjects, this must be explained in the consent process, particularly in the alternatives to participation section.

Discussion Notes

Reviewers discussed that the age limit (49) is likely because proprioception decreases with age. However, the applicant could have defined exclusion more precisely, not simply as age.

Regulatory Strategy and Transition Plan
Average Score: 7.4

Scientist Reviewer A

Strengths: All devices and products proposed to be used as part of the study are FDA approved and marketed in the United States.

Weaknesses: No weaknesses were noted.

Scientist Reviewer B

Strengths: The application includes documentation that the study is exempt from FDA regulation. The clinical protocol being investigated will be disseminated through normal research avenues. The PI has a schedule and milestones in place for the 3 aims proposed in the study and dissemination of the research findings in relevant conferences/journals. The PI aligns well with the clinicians/researchers, who have similar interests and plans to pursue funding for future clinical trials. No intellectual property is being developed as part of this project.

Weaknesses: No risk analyses for cost, schedule, or sustainability are included. Clearer milestones for continued development would strengthen the transition plan.

Regulatory Compliance Specialist Reviewer

Strengths: The regulatory plan appears appropriate given that the treadmill to be utilized is commercially available and is being used for its intended purpose. No additional FDA oversight or clearance is required.

Procurement Sensitive Document
Do not copy or distribute without CDMRP written permission.

The transition plan appears appropriate as the knowledge products to be developed are well described and there is a plan to implement knowledge transfer across a number of outlets, for example, grand rounds at a range of military, veteran, and civilian hospitals. Given the positions held and relationships developed by the investigators of this proposal, it appears that there will be no issue disseminating the knowledge gained from this project and integrating it into standard practice.

Knowledge products in the form of publications will be written with the intent to impact the next clinical practice guidelines. Presentation of the findings will occur at national conferences and scientific meetings. Two investigators are members of the Extremity Trauma and Amputation Center of Excellence. Another investigator is a member of the Clinical Affairs Division's Knowledge Translation Committee, which works together to ensure that research results are translated to clinicians in a useful manner. Additional knowledge transfer will occur through clinician training and patient education.

The study team may pursue a phase 4 clinical trial to further the research results. If the results are promising, the study team may target other patient populations to promote safe return to duty or sports participation. There are no plans to generate intellectual property.

Weaknesses: The Technical Abstract indicates the study design is a parallel randomized clinical trial with patients randomly assigned to treatment (n = 58) or control (n = 58). However, the Lay Abstract notes that 312 will participate, with half in the treatment and half in control.

Personnel and Communication
Average Score: 6.8

Scientist Reviewer A

The PI, Elizabeth Russell Esposito, received a PhD in kinesiology from the University of Massachusetts, Amherst, Massachusetts, in 2011. The PI is currently an assistant professor, Uniformed Services University, Bethesda, Maryland; a senior scientist, Center for the Intrepid, Fort Sam Houston, Texas; and an affiliate assistant professor, Dept Mechanical Engineering, University of Washington.

Strengths: The PI and the Coinvestigators appear to have significant experience in implementing a clinical trial.

Weaknesses: The proposal would be improved with adding a consultant with expertise in motor control/proprioception.

Scientist Reviewer B

Strengths: The PI's background is well suited to perform this research. The PI has a strong publication record. The team the PI has put together for this project is strong and has the expertise. The composition of the team is good; the investigators have the experience to complete this project. The team members are all productive, and their skills complement each other very well. The project management and multisite communication plan look adequate. Each is devoting appropriate effort to the proposed project. There is adequate evidence of institutional support.

Weaknesses: The PI has limited published work in people with CAI and limited experience with this patient population.

Procurement Sensitive Document
Do not copy or distribute without CDMRP written permission.

UNSCORED CRITERIA

Environment

Scientist Reviewer A

The environment is well described and seems to be appropriate for this study. Since participants are in the military, recruitment and avoiding attrition are improved.

Scientist Reviewer B

The research environment and the facilities at the VA Puget Sound Health Care System, Madigan Army Medical Center, Naval Health Research Center, and Naval Medical Center San Diego seem to be suited to the project and its timely completion. The PI has obtained necessary letters of support. The infrastructure, resources, and collaborators are of high caliber. There is evidence of appropriate institutional support.

Budget

Scientist Reviewer A

The budget is clear. Regarding equipment, electromyography (EMG) and a Plantar pressure system are budgeted. It may be assumed, but is not clearly stated, that this equipment is available in San Diego but that this equipment is needed for the sites in Washington State.

Scientist Reviewer B

The duration of the study (4 years) seems appropriate. Also, funding is requested to buy few equipment, but no quote has been submitted with the proposal.

Biostatistician Reviewer

A biostatistician is included at 5% per year. This is appropriate at the beginning of the study; however, due to the complexity and amount of analyses required in the last year or so, this percentage is quite modest.

Application Presentation

Scientist Reviewer A

This application is very clear, and the study design is appropriate. The application is well written.

Scientist Reviewer B

The application is complete and logically organized. However, there are some inadequate explanations, undefined terms, formatting issues, and typographical errors in the proposal.

Biostatistician Reviewer

Figures greatly enhance the application presentation. The writing is clear, and important facts are reinforced with relevant citations.

Procurement Sensitive Document
Do not copy or distribute without CDMRP written permission.
